# Fermentability of Novel Type-4 Resistant Starches in In Vitro System

**DOI:** 10.3390/foods7020018

**Published:** 2018-02-01

**Authors:** Jennifer M. Erickson, Justin L. Carlson, Maria L. Stewart, Joanne L. Slavin

**Affiliations:** 1 Food Science and Nutrition Department, University of Minnesota, 1334 Eckles Ave, St. Paul, MN 55108, USA; eric2472@umn.edu (J.M.E.); carl2814@umn.edu (J.L.C.); 2 Ingredion Incorporated, 10 Finderne Ave, Bridgewater, NJ 08807, USA; maria.stewart@ingredion.com

**Keywords:** prebiotics, resistant starch, dietary fiber

## Abstract

Resistant starches are non-digestible starches that are fermented in the colon by microbiota. These carbohydrates are prebiotic and can be beneficial to consumer health. Many types of resistant starch exist with varying physical properties that may result in differences in fermentability. The objective of this research project was to compare potential prebiotic effects and fermentability of four novel resistant starches using an in vitro fermentation system and measuring changes in total gas production, pH, and formation of SCFAs (short chain fatty acids). Fecal donations were collected from seven healthy volunteers. Four novel resistant starches, modified potato starch (MPS), modified tapioca starch (MTS), and modified maize starches (MMS-1 and MMS-2), were analyzed and compared to polydextrose and short chain fructooligosaccharides (FOS) as controls. After twenty-four hours of fermentation, MPS and MTS responded similarly in gas production (74 mL; 70.6 mL respectively), pH (5.93; 5.93 respectively), and SCFA production (Acetate: 115; 124, Propionate: 21; 26, Butyrate: 29; 31 μmol/mL respectively). While MMS-1 had similar gas production and individual SCFA production, the pH was significantly higher (6.06). The fermentation of MMS-2 produced the least amount of gas (22 mL), with a higher pH (6.34), and lower acetate production (78.4 μmol/mL). All analyzed compounds were fermentable and promoted the formation of beneficial SCFAs.

## 1. Introduction

The importance of dietary fiber for human health is well documented [1], yet average fiber consumption in the United States is well below the recommended Adequate Intake of 25 g/day for women and 38 g/day for men [2]. The addition of dietary fiber to food products may be an effective way to increase fiber intake in consumers. In 2016, the Food and Drug Administration (FDA) announced a change in the definition of fiber beyond simply non-digestible carbohydrates of three or more monosaccharide units long [3]. The FDA has proposed that isolated and synthetic non-digestible carbohydrates must also have a proven physiologic benefit to human health to be considered and labeled as dietary fiber [3]. Resistant starches are among several non-digestible carbohydrates being considered as dietary fiber sources by the FDA [4]. Resistant starches are starches that are not able to be digested by enzymes in the small intestine and are fermented in the colon by microbiota [5]. While resistant starches can be found naturally in food products like less-ripe bananas, legumes and potatoes [6], there are many different types of resistant starches developed through starch modification for use in food production. 

Resistant starches are a valuable ingredient solution for product developers looking to enhance the fiber content of food products. Resistant starches have a mild flavor, are naturally white in color, and can be made either soluble or insoluble in water [7]. When added to food products in place of traditional fibers, resistant starches can produce a better appearance, texture and mouthfeel [8]. Baixauli et al. substituted up to 20% resistant starch for traditional starch in a baked application with no significant difference in taste or acceptability [9]. Because of their functional properties, resistant starches are able to be added in a variety of food applications including dairy products, baked goods, and battered goods [7]. 

Beyond their sensory acceptability, consumption of resistant starches has several health benefits, including a reduced glycemic response [10], improved blood lipid profile [11], and potentially increased satiety response [12,13]. Additionally, resistant starches are prebiotic, promoting the growth of beneficial bacteria in the colon through the fermentation process [6]. The prebiotic nature of resistant starches results in the production of gas and short chain fatty acids (SCFA), which can result in a lowered colonic pH. Gas production is a byproduct of the fermentation process, and large amount of gas can result in gastrointestinal discomfort and flatulence in some individuals [14]. The production of SCFAs are beneficial for the host’s health, as they lower the colonic pH which has been associated with reduced pathogenic bacteria in hindgut [15,16], improve mineral absorption [17,18,19], and reduced risk of colon cancer [6]. Additionally, SCFA can be used as an energy source for the host [20]. SCFA production varies greatly between hosts, and depends largely on sources for fermentation [21].

As our understanding of prebiotics and their effects on human health grows, it is important to examine the individual effects and differences between types of prebiotics that may be used in food applications. Because there are many different types of modified starches with different functional properties, knowing the unique effect of the individual types of resistant starch is necessary. This paper aims to compare the fermentability of four novel resistant starches (type 4 resistant starch, RS4) using an in vitro fermentation system measuring changes in total gas production, pH, and formation of common SCFAs. Type 4 resistant starches, or RS4, are a class of starches that have been modified through a chemical process that renders the starch indigestible [7]. A rapidly fermented carbohydrate control and a slowly fermented carbohydrate control were also measured for comparison. 

## 2. Materials and Methods 

### 2.1. Resistant Starches Analyzed 

Four resistant starches were chosen for this study (Table 1), including modified potato starch (MPS), modified tapioca starch (MTS), and modified maize starches (MMS-1 and MMS-2), which were compared to polydextrose, a slowly fermented carbohydrate control, and short chain fructooligosaccharides (FOS), a rapidly fermented carbohydrate control. The samples were provided by Ingredion Incorporated (Bridgewater, NJ, USA). The polydextrose was obtained from Shandong Bailong Chuangyuan Bio-Tech Co., Ltd. (Yucheng, Shandong, China). All of the starches are food grade modified starches. The distarch phosphates (MPS, MTS, MMS-2) comply with the Joint Food and Agriculture Organization and World Health Organization Expert Committee on Food Additives standards for phosphate content [22]. 

### 2.2. Predigestion

Predigested samples were prepared via enzymatic digestion modeled on the total dietary fiber method (AOAC 991.43). The procedure was performed with 350 g at 39% solids to enable mixing. Samples were digested with 17.5 mL thermostable α-amylase Termamyl^®^ 120 L, (Novozymes, Bagsvaerd, Denmark), Type L in buffer (MES pH = 8.2 at 24 °C) at 90–95 for 35 min. then cooled to 60 °C, digested with 35 mL protease Multifect^®^ PR6L (Dupont, Wilmington, DE, USA) for 30 minutes, followed by pH adjustment to 4.4–4.7 and digestion with 70 mL amyloglucosidase Optidex^®^ L-400 (Dupont, Wilmington, DE, USA) at 60 °C for 30 min. Reaction samples were precipitated in ethanol for 1 h, recovered by filtration and air drying overnight. The dried samples were ground to pass through a 60 mesh screen (<250 microns).

### 2.3. Fecal Collection and Donor Information

Fecal samples were collected from seven healthy volunteers (4 males, 3 females, ages 21–28) consuming non-specific Western diets, free of any antibiotic treatments in the last year, non-smokers, not affected by any known gastrointestinal diseases and not consuming any supplements (Table 2). Volunteers were screened using a brief dietary questionnaire assessing their typical fiber consumption. Individuals consuming a high-fiber diet (consuming three or more servings of high-fiber foods on 4 or more days per week) or any fiber supplements were excluded. Fecal samples were anaerobically collected within 5 min of the start of the fermentation using a specimen collection device (Medline Specimen Collection Kit, Medline, Inc., Rogers, MN, USA), and homogenized immediately upon collection. All data and samples collected were done in accordance with University of Minnesota policies and procedures. The number of fecal donors utilized in the study was based on the maximum number of samples able to be run with the in vitro model given the constraints of the equipment and environment. Coles et al. recommends five or more donors to minimize the effect of individual variation [23]. 

### 2.4. Consent

Voluntary informed consent was obtained from all fecal donors prior to this study, in accordance with University of Minnesota policies and procedures. 

### 2.5. Fermentation

Fiber samples (0.5 g) were hydrated in 40 mL of prepared sterile trypticase peptone fermentation media in 100 mL serum bottles, capped, and incubated for 12 h at 4 °C. Following incubation, serum bottles were transferred to a circulating water bath at 37 °C and allowed to incubate for 2 h. Post-collection, fecal samples were mixed using a 6:1 ratio of phosphate buffer solution to fecal sample. After mixing, obtained fecal slurry was combined with prepared reducing solution (2.52 g cysteine hydrochloride, 16 mL 1 N NaOH, 2.56 g sodium sulfide nonanhydride, 380 mL DD·H_2_O) at a 2:15 ratio. 10 mL of the prepared fecal inoculum was added to each of the serum bottles, 0.8 mL Oxyrase^®^ (Oxyrase, Inc., Mansfield, OH, USA) was added, flushed with CO_2_, sealed, and then immediately placed in a 37 °C circulating water bath. Fecal donations were measured for gas, pH and SCFA at baseline. Samples were prepared in triplicate and analyzed at 4, 8, 12 and 24 h. Upon removal at each time point, total gas volume was measured. Then samples were divided into aliquots for analysis and 1 mL of copper sulfate (200 g/L) was added to cease fermentation. All samples were immediately frozen and stored at −80 °C for further analysis.

### 2.6. Gas Analysis

Total gas production was measured by syringe difference analysis. The cap of each serum bottle was punctured with a syringe needle, allowing the gas to be released into a syringe. Gas released was collected and measured for each of the sealed serum bottles. Measurements were corrected for baseline values. 

### 2.7. pH Analysis

Sample pH values were measured with the 350 PerpHect LogR meter (Thermo Fisher Scientific Inc., Waltham, MA, USA) following the provided operating instructions. 

### 2.8. SCFA Analysis

SCFA samples were extracted according to Schneider et al. [24] with minor modifications. Small samples (2 mL) were removed from the −80 °C freezer and thawed at 4 °C for 12 h prior to SCFA analysis. Samples were vortexed for 5 s, then 1.6 mL of deionized H20, 400 μL H_2_SO_4_ (50% vol/vol), and 2 mL diethyl ether (premixed with 2-ethyl butyric acid as internal standard) were combined with the sample, and the tubes were vortexed again for another 5 s. An orbital shaker was utilized for 45 min at 100 revolutions per minute (RPM). The sample mixtures were then centrifuged for 5 min at 3000 RPM. Supernatant was removed from tube and placed in 10 mL plastic tubes containing CaCl_2_ to remove any residual water. The solution was then filtered using a BD 1 mL syringe (Becton, Dickinson and Company, Franklin Lakes, NJ, USA) and a Millex 13 mm nylon membrane filter with a 0.20 μm pore size (Merck Millipore Ltd. Tullagreen, Carrigtwohill, Co. Cork, Ireland). Extractions were then analyzed using a HP 5890 series gas chromatograph (Hewlitt Packard, Palo Alto, CA, USA) with a 30 m × 0.250 mm × 0.25 μm polyethylene glycol (PEG) column (Agilent Technologies, Santa Clara, CA, USA), with a 110 °C oven temperature. Samples were injected using an automated HP 7673 GC/SFC injector (Hewlitt Packard, Palo Alto, CA, USA). Injector and detector temperatures were 220 and 240 °C, respectively. Flow rates for air, helium and hydrogen were 26, 28 and 315 mL/min, respectively. All samples were analyzed with a 50:1 split ratio. These methods have been used to extract and analyze samples in previous studies by the authors [25,26,27]. Measurements were corrected for baseline values.

### 2.9. Statistical Analysis

One-way analysis of variance (ANOVA) was conducted on pH, gas, total SCFA and individual SCFAs at each time point using GraphPad Prism 7 (v 7.03, GraphPad Software, Inc., La Jolla, CA, USA). Tukey’s pair-wise comparison was conducted on each time point to identify significant differences among fibers, *p* values <0.05 were deemed statistically significant.

## 3. Results

### 3.1. Gas Production

**Figure 1:**
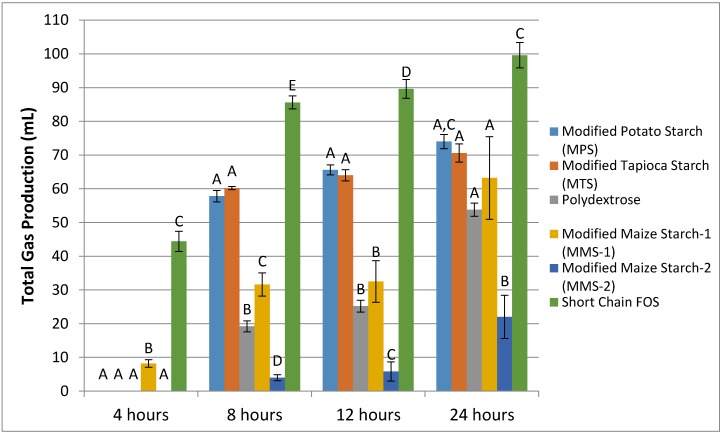
Mean total gas production following the in vitro fermentation of various carbohydrate sources at between 4 and 24 h post exposure to fecal microbiota of five different donors. Error bars indicate standard error. Columns with different letters are significantly different from one another within each time measurement. *n* = 15 (5 donors × 3 replicates), FOS: fructooligosaccharides.

**Figure 2:**
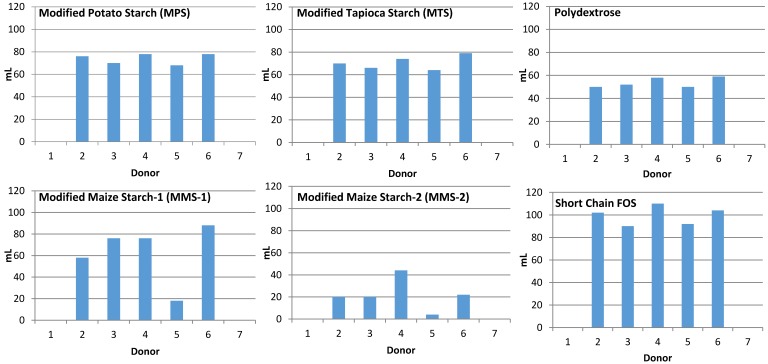
Total gas production after 24 h of fermentation of various carbohydrate sources in an in vitro system for each individual donor. Blank spaces above a donor number indicate a sample that did not produce gas during the in vitro fermentation.

Samples from non-gas producing donors (*n* = 2) were excluded from analysis. The fermentation of MPS and MTS treatments resulted in similar mean gas production at each time point (Figure 1). Fermentation of MMS-2 resulted in the lowest mean gas production. Fermentation of short chain FOS produced more gas than any of the other carbohydrates tested, at each time point (*p* < 0.05). At 24 h, the MMS-2 produced significantly less gas than any of the other tested carbohydrates (*p* < 0.02), producing less than half the amount of gas as the next lowest gas producing carbohydrate, polydextrose and 4.5 times less than the gas produced from the short chain FOS treatment. While the difference in mean gas production between MPS and short chain FOS was insignificant (*p* = 0.063), the difference in mean gas production was significant between short chain FOS and MTS (*p* = 0.027), polydextrose (*p* < 0.001), MMS-1 (*p* = 0.004) and MMS-2 (*p* < 0.0001) at 24 h. Individual gas production for each donor at 24 h is presented in Figure 2. 

### 3.2. pH Production

**Figure 3:**
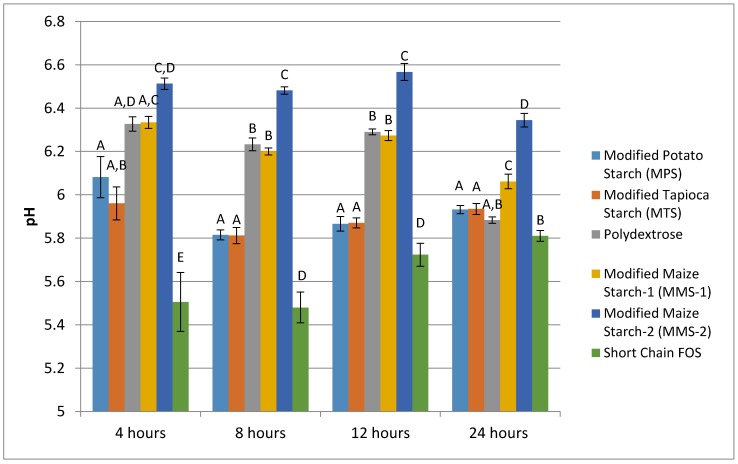
Mean pH of various carbohydrate sources following in vitro fermentation between 4 and 24 h post exposure to fecal microbiota of seven different donors. Error bars indicate standard error. Columns with different letters are significantly different from one another within each time measurement. *n* = 21 (7 donors × 3 replicates).

At each time point measured, the fermentation of short chain FOS produced the lowest mean pH, and MMS-2 produced the highest mean pH values (Figure 3). The pH values ranged from 5.51 to 6.51 at 4 h, 5.48 to 6.48 at 8 h, 5.72 to 6.57 at 12 h, and 5.81 to 6.34 at 24 h of fermentation. The fermentation of MPS and MTS resulted in similar pH response at each time point. The fermentation of MMS-2 and polydextrose was similar at 4, 8 and 12 h post fermentation; however, at the 24 h time point, the mean pH values for the two treatments were statistically different (*p* < 0.001). At 24 h, the mean pH of polydextrose was similar to MPS (*p* = 0.760), MTS (*p* = 0.715) and short chain FOS (*p* = 0.356). All other pairwise comparisons of the various carbohydrates tested resulted in statistically significant differences in pH values at the 24 h time point.

### 3.3. SCFA Production

Analysis of SCFA shows production of SCFA from baseline corrected samples at 12 and 24 h. SCFA production is measured in μmol/mL of fermentation media, and presented as the mean value for each treatment and time point. 

**Figure 4:**
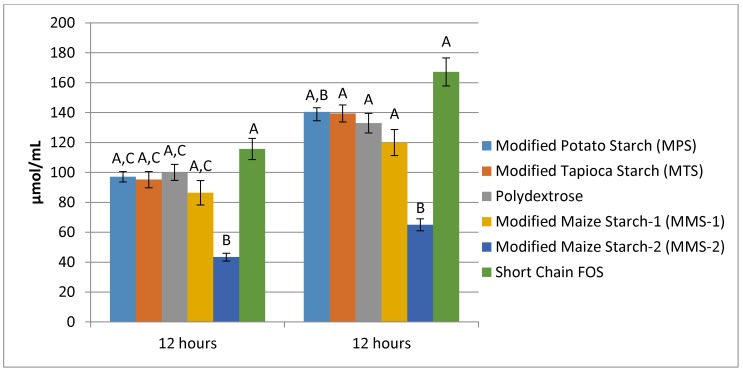
Mean acetate production at 12 and 24 h of fermentation in an in vitro system with various carbohydrate sources. Error bars indicate standard error. Columns with different letters are significantly different from one another within each time measurement. *n* = 21 (7 donors × 3 replicates).

At 12 h of fermentation, the mean acetate produced ranged from 43.41 μmol/mL in the MMS-2 samples to 115.71 μmol/mL in the short chain FOS samples. Acetate production was similar for the MPS, MTS, Polydextrose, MMS-1 and short chain FOS at 12 h of fermentation (Figure 4). The fermentation of MMS-2 produced a significantly different mean acetate when compared to all other fibers (*p* < 0.05), resulting in the production of less than half the amount of acetate compared to the other tested carbohydrates. Additionally, the difference in mean acetate production between MMS-1 and short chain FOS was also significantly different (*p* < 0.01). At 24 h, the mean acetate production ranged from 78.36 μmol/mL in the MMS-2 samples to 149.68 μmol/mL in the short chain FOS samples. Acetate production following 24 h of fermentation was significantly different in MMS-2 when compared to MTS (*p* = 0.023), Polydextrose (*p* < 0.001), MMS-1 (*p* = 0.003) and short chain FOS (*p* < 0.001).

**Figure 5:**
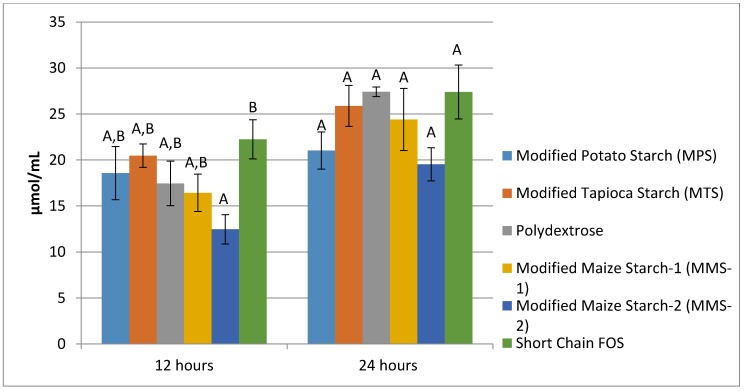
Mean propionate production at 12 and 24 h of fermentation in an in vitro system with various carbohydrate sources. Error bars indicate standard error. Columns with different letters are significantly different from one another within each time measurement. *n* = 21 (7 donors × 3 replicates).

Mean propionate production at 12 h was similar among MPS, MTS, Polydextrose, and MMS-1 (Figure 5). The fermentation of short chain FOS produced the greatest amount of propionate (22.24 μmol/mL), while the fermentation of MMS-2 produced the least amount (12.46 μmol/mL) at 12 h. There was a significant difference in mean propionate production between the MMS-2 samples and the short chain FOS samples at 12 h (*p* = 0.03). At 24 h, each of the test carbohydrates produced statistically similar mean propionate values, ranging in amounts from 19.52 to 27.41 μmol/mL. 

**Figure 6:**
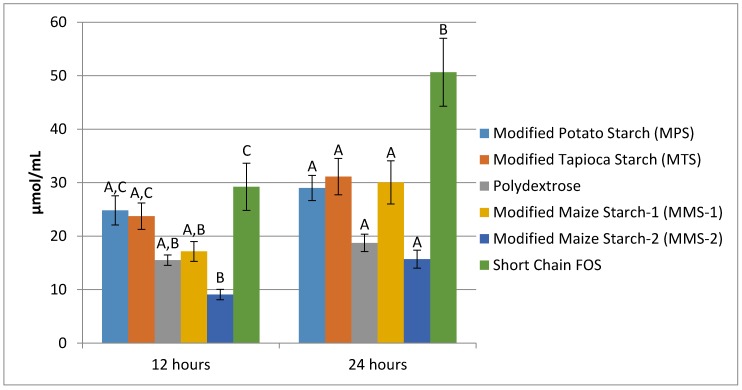
Mean butyrate production at 12 and 24 h of fermentation in an in vitro system with various carbohydrate sources (*n* = 7). Error bars indicate standard error. Columns with different letters are significantly different from one another within each time measurement. *n* = 21 (7 donors × 3 replicates).

Mean butyrate production following 12 h of fermentation ranged from 9.07 μmol/mL in the MMS-2 sample to 29.23 μmol/mL in the short chain FOS sample (Figure 6). The fermentation of MMS-2 produced significantly different amounts of butyrate compared to the MPS (*p* = 0.001), MTS (*p* = 0.004) and short chain FOS (*p* < 0.001) at 12 h. Additionally, the fermentation of the short chain FOS samples produced significantly different amounts of butyrate compared to polydextrose (*p* = 0.006) and MMS-1 (*p* = 0.019). Mean butyrate production at 24 h of fermentation ranged from 15.69 μmol/mL (MMS-2) to 50.65 μmol/mL (short chain FOS). After 24 h of fermentation, the amount of butyrate produced from the short chain FOS samples was significantly different from all other carbohydrates tested (*p* < 0.05), producing more than three times more butyrate compared to the MMS-2 sample.

**Figure 7:**
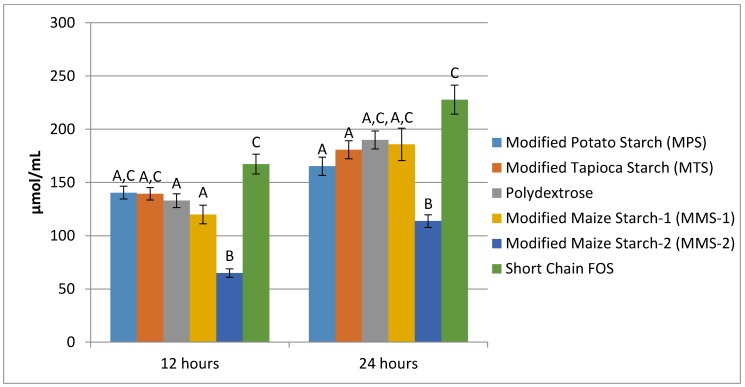
Mean total SCFA production at 12 and 24 h of fermentation in an in vitro system with various carbohydrate sources (*n* = 7). Error bars indicate standard error. Columns with different letters are significantly different from one another within each time measurement. *n* = 21 (7 donors × 3 replicates).

Mean total SCFA production after 12 h ranged from 64.93 μmol/mL for the MMS-2 treatment to 167.18 μmol/mL in the short chain FOS treatment (Figure 7). The fermentation of short chain FOS resulted in significantly different total SCFA production compared to polydextrose (*p* < 0.015) and MMS-1 (*p* < 0.001). Mean total SCFA production at 24 h ranged from 113.83 μmol/mL for MMS-2 to 227.73 μmol/mL for short chain FOS. The fermentation of short chain FOS resulted in a significantly different total SCFA production compared to MPS (*p* < 0.002) and MTS (*p* < 0.0475) after 24 h of fermentation. The total SCFA produced during the fermentation of MMS-2 was significantly less than all of the other carbohydrate treatments at both 12 and 24 h (*p* < 0.05). The short chain FOS treatment resulted in the greatest total SCFA production, producing more than double the amount of SCFAs compared to the MMS-2 treatment after both 12 and 24 h of fermentation. 

**Figure 8:**
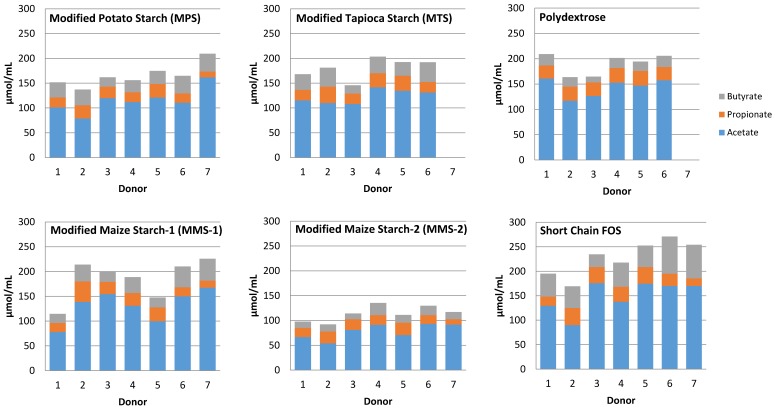
Total SCFA production after 24 h of fermentation of various carbohydrate sources in an in vitro system for each individual donor.

Individual variability was observed in response to each carbohydrate treatment resulting in varied amounts of SCFA produced (Figure 8). Responses between treatments showed lowest total SCFA production from the MMS-2 treatment for each donor. However, some donor inoculum responded differently when exposed to different treatments. The mean total SCFA production at 24 h was greatest for the short chain FOS treatment (Figure 6), but the inoculum from Donor 1 produced the most total SCFA when fermented with polydextrose and the inoculum from Donor 2 produced the most total SCFA when fermented with MMS-1. All other donors’ inoculum produced the greatest total SCFA with the short chain FOS treatment. 

## 4. Discussion

The aim of this study was to examine the effects of the fermentation of various novel resistant starches, including the production of, gas, change in pH, and SCFA production. It is important to note that while this study attempts to mimic the physiologic effects that fibers and resistant starches would have within the gastrointestinal system, this study was done in vitro, and we can never truly replicate the complex effects that the test substances would have in vivo. This is a limitation of all in vitro study designs, and the results should be considered with this in mind. Future research should include a study in vivo to better understand the physiologic effects resulting from the supplementation of these novel resistant starch sources in the diet. 

Gas production increased as a function of time for all carbohydrates measured in this in vitro study. Large amounts of gas production in vivo can result in gastrointestinal discomfort, particularly in populations with visceral hypersensitivity, such as individuals with irritable bowel syndrome [14,28]. Due to the nature of the study, only total gas produced was able to be measured, and the ability to distribute and eliminate the gas in vivo is unknown. Understanding how the carbohydrates influence not only gas production, but also gas retention is important to better understand how these carbohydrates influence gastrointestinal tolerance [14]. We would expect an increase in gas production to also produce more flatulence to manage the increased gas occurrence. Some individuals may not be able to create such equilibrium, and may experience gastrointestinal distress as a result of carbohydrates that produce large amounts of gas. Short chain FOS, which has been documented to result in gastrointestinal intolerance when consumed in large amounts [29], resulted in the greatest gas production over 24 h of fermentation. MMS-2 produced the least amount of gas (22 mL after 24 h of fermentation), and may be better tolerated, especially in individuals with visceral hypersensitivity. 

With the exception of the short chain FOS, the fermentation of each of the test carbohydrates resulted in a decrease in pH between the 4 and 24 h time point. At 24 h, the short chain FOS treatment resulted in the lowest pH of all the test carbohydrates. However, the pH of the short chain FOS actually increased in pH between the time points of 4 h and 24 h of fermentation. This result is not understood; however, a similar study previously found an increase in pH between 12 and 24 h during the in vitro fermentation of inulin [30]. Decreased pH is an expected result of the fermentation process, and is due to the SCFA production, which lowers the pH of the surrounding environment [31]. Lower colonic pH can have beneficial effects for the host’s health. A lower pH may reduce the amount of pathogenic bacteria in the intestine without influencing the quantity of bifidobacteria [15,16], increase the rate of mineral absorption of minerals like calcium [17,18,19], and may reduce the risk of colon cancer [32,33,34]. 

The fermentation of each test carbohydrate produced the individual SCFAs acetate, propionate and butyrate. Although there was no difference in propionate production at 24 h, short chain FOS produced significantly more butyrate compared to the other test carbohydrates, while MMS-2 produced significantly less acetate compared to MTS, polydextrose, MMS-1 and short chain FOS. Beyond the pH-lowering effects of SCFA production described above, SCFA can be an energy source for the host, providing up to 10% of metabolizable energy [20]. SCFA production varies greatly between hosts, and depends largely on sources for fermentation [21]. 

Individual variability of the donor’s microbiome can play a major role in the fermentation response of the resistant starches [35]. This is particularly evident with respect to gas production in the present study (Figure 2). Although the same procedures were used, the bacteria from some of the donors produced considerable gas, while samples from other donors did not produce any measurable gas. There was also variability in the SCFA production (Figure 8) among the different donors. Some donors were able to ferment a fiber more efficiently than other donors. Additionally, there was individual variation within each donor’s ability to ferment the various types of fibers. For example, the inoculum from Donor 1 was able to produce more SCFA after fermentation of Polydextrose and short chain FOS, compared to MMS-1 or MMS-2 (Figure 8). This varied fermentation response to the samples is reflective of the variability of responses that can occur when non-digestible carbohydrates are consumed. A recent study found that the supplementation of resistant starches in young adults increased butyrate production in some participants, but did not significantly increase in other participants [35]. Venkataraman et al. hypothesized that a low abundance of certain bacteria known to break down resistant starches may be the cause of the limited response to the resistant starch supplementation [35]. Therefore, personalized dietary interventions may be appropriate to facilitate the greatest beneficial health effects based on the individual’s microbiome. More research is needed to assess the individual variability of the microbiome to better understand these effects. The present study is limited in its results, as fecal samples from only seven donors were used. A larger sample size may have provided a more representative view of the fermentation effects of the tested resistant starches. Additionally, the donor’s diets were not controlled in the days prior to donation. The diet of the donors in the days prior to the study could have impacted the results.

## 5. Conclusions

The tested carbohydrates each had unique responses to the fermentation, resulting in differences in gas production, change in pH, and SCFA production. After twenty-four hours of fermentation, MPS and MTS responded similarly in all aspects, while MMS-1 had similar gas production and SCFA production, and the pH was significantly higher. The fermentation of MMS-2 produced the least amount of gas, with significantly higher pH levels and less acetate production. Overall, each of the novel resistant starches tested in this in vitro fermentation study proved to be fermentable, and promoted the production of SCFA. However, some treatments were more responsive than others. MMS-1 produced a total SCFA concentration statistically similar to FOS after 24 h of fermentation. Beyond SCFA production, the fermentation of MMS-1 resulted in a lower total gas production than short chain FOS at all time points. As a result, MMS-1 may be a good candidate to replace FOS in some applications, particularly for consumers who may be concerned about gastrointestinal tolerance. Additionally, MPS and MTS had similar fermentation responses to polydextrose, and could be a suitable substitute in food applications. The consumption of these novel resistant starches likely plays a role in the physiologic response of the consumer. Studies should be conducted in vivo to further assess the prebiotic effects of these novel resistant starches. 

## Figures and Tables

**Table 1:** Comparison of resistant starches and controls analyzed with in vitro fermentation system.

Carbohydrate Source	Sample Tradename, Chemical Characterization (If Applicable)	Grams of Fiber/100 g	Grams of Starch/100 g	Grams of Sugar/100 g
Modified potato starch (MPS)	VERSAFIBE^TM^ 1490 dietary fiber, Distarch phosphate	74.2	11.8	0
Modified tapioca starch (MTS)	NOVELOSE^TM^ 3490 dietary fiber, Distarch phosphate	79.9	7.9	0
Modified maize starch (MMS-1)	VERSAFIBE^TM^ 2470 dietary fiber, Acid hydrolyzed and heat treated	65	24.9	0
Modified maize starch (MMS-2)	VERSAFIBE^TM^ 2480 dietary fiber, Distarch phosphate	71	14	0
Polydextrose	n/a	87.5	0	4.5
Short-chain fructooligosaccharide	NUTRAFLORA^®^ soluble prebiotic fiber	92.5	0	4.3

**Table 2:** Demographic Characteristics of Seven Fecal Donors.

	Donor 1	Donor 2	Donor 3	Donor 4	Donor 5	Donor 6	Donor 7
Sex	Female	Male	Male	Male	Female	Male	Female
Age	21	21	23	27	25	28	28
BMI	20.0	30.1	24.0	25.2	17.7	27.7	25.8

BMI: Body Mass Index.
